# Effects of Brief Mental Skills Training on Emergency Medicine Residents’ Stress Response During a Simulated Resuscitation: A Prospective Randomized Trial

**DOI:** 10.5811/westjem.2021.10.53892

**Published:** 2022-01-03

**Authors:** Matthew Aronson, Timothy Henderson, Kenneth W. Dodd, Michael Cirone, Margaret Putman, David Salzman, Elise O. Lovell, Kelly Williamson

**Affiliations:** * Advocate Christ Medical Center, Department of Emergency Medicine, Oak Lawn, Illinois; † Northwestern Medicine, Department of Anesthesia/Critical Care, Chicago, Illinois; ‡ Swedish American Hospital, Department of Emergency Medicine, Rockford, Illinois; § University of Illinois at Chicago, Department of Emergency Medicine, Chicago, Illinois; ¶ Hennepin County Medical Center, Department of Medicine, Minneapolis, Minnesota; || Northwestern University, Feinberg School of Medicine, Department of Emergency Medicine and Department of Medical Education, Chicago, Illinois; # Northwestern University, Feinberg School of Medicine, Department of Emergency Medicine, Chicago, Illinois

## Abstract

**Background:**

Acute stress impairs physician decision-making and clinical performance in resuscitations. Mental skills training, a component of the multistep, cognitive-behavioral technique of stress inoculation, modulates stress response in high-performance fields.

**Objective:**

We assessed the effects of mental skills training on emergency medicine (EM) residents’ stress response in simulated resuscitations as well as residents’ perceptions of this intervention.

**Methods:**

In this prospective, educational intervention trial, postgraduate year-2 EM residents in seven Chicago-area programs were randomly assigned to receive either stress inoculation training or not. One month prior to assessment, the intervention group received didactic training on the “Breathe, Talk, See, Focus” mental performance tool. A standardized, case-based simulation was used for assessment. We measured subjective stress response using the six-item short form of the Spielberger State-Trait Anxiety Inventory (STAI-6). Objective stress response was measured through heart rate (HR) and heart rate variability (HRV) monitoring. We measured subjects’ perceptions of the training via survey.

**Results:**

Of 92 eligible residents, 61 participated (25 intervention; 36 control). There were no significant differences in mean pre-/post-case STAI-6 scores (−1.7 intervention, 0.4 control; *p* = 0.38) or mean HRV (−3.8 milliseconds [ms] intervention, −3.8 ms control; *p* = 0.58). Post-assessment surveys indicated that residents found this training relevant and important.

**Conclusion:**

There was no difference in subjective or objective stress measures of EM resident stress response after a didactic, mental performance training session, although residents did value the training. More extensive or longitudinal stress inoculation curricula may provide benefit.

## INTRODUCTION

Emergency medicine (EM) residents often encounter highly stressful clinical situations and must perform life-saving interventions with limited time, resources, and background information. Resuscitation requires a rapid and dynamic integration of numerous cognitive processes including information-gathering, communication, decision-making, and physical skills.^1^ It has been demonstrated that high levels of acute stress and anxiety can critically impair physician decision-making.^2^ Additionally, high levels of perceived stress have been shown to impair healthcare professionals’ clinical performance in acute resuscitation scenarios.^3^–^5^ Other high-stress realms such as the military, aviation, athletics, and business have adopted and cultivated mental skills techniques to enhance performance under pressure; however, similar programs have been notably absent in medical training.^6^,^7^

Stress inoculation, a multistep, cognitive behavioral technique, has been demonstrated to be effective across numerous high-performance domains.^8^–^11^ Stress inoculation involves three phases: 1) learning about the effects of acute stress on performance; 2) acquiring and rehearsing specific mental skills and coping strategies to optimize performance under stress; and 3) applying these skills and strategies to real-world, high-stress environments.^8^ Mental skills training has been shown to enhance performance and coping in stressful situations in pilots, police, military special forces, professional athletes, and surgeons.^12^–^16^ While resident physicians frequently encounter stressful clinical resuscitations, there are no formal Accreditation Council for Graduate Medical Education (ACGME) recommendations for educating resident physicians about the impacts of acute stress on performance; nor is there mention of the potential role of mental skills training to optimize performance under stress.

While assessing EM residents during clinical patient resuscitations is desirable, simulation provides a controlled and reproducible environment for examination of variables that contribute to both objective and subjective stress as well as assessment of clinical performance.^17^ Simulated resuscitation scenarios have been demonstrated to elicit high levels of subjective and objective measures of stress in EM residents, allowing extrapolation of findings from simulation to clinical medicine.^18^

In this study our aim was to assess the implementation of a brief mental skills training on perceived and actual stress in EM residents during simulated resuscitation scenarios. We hypothesized that the intervention group would have less subjective stress as measured by pre- and post-simulation scores on the six-item, short form of the Spielberger State-Trait Anxiety Inventory (STAI-6).^19^,^20^ Secondary outcomes included physiologic measures of stress (ie, heart rate [HR] and heart rate variability [HRV]) and residents’ perception of the stress- inoculation skills training program.

## METHODS

### Study Design

This study was a multicenter prospective educational trial performed at seven ACGME-accredited EM residency programs in Chicago, Illinois. The study was reviewed and approved by each institution’s institutional review board.

### Subjects

Eligible subjects for this study were postgraduate year (PGY)-2 EM residents at the participating programs during the study period January–February 2020. Participation in the study was voluntary. Informed consent was obtained from all subjects. Based upon prior studies, we anticipated a 20% difference in STAI-6 scores between groups. Power analysis, with power 0.8 and alpha 0.05, yielded a minimum of 36 subjects needed in each group.

**Population Health Research Capsule**

What do we already know about this issue?

*Acute stress impairs physician decision-making and clinical performance in resuscitations. Emergency medicine (EM) residents often encounter highly stressful clinical situations*.

What was the research question?

*We studied the effects of mental skills training on EM resident stress response in simulated resuscitations, as well as resident perceptions of this intervention*.

What was the major finding of the study?

*There was no difference in subjective or objective stress measures of EM resident stress response after a didactic mental performance training session, although residents did value the training*.

How does this improve population health?

*High levels of acute stress and anxiety can critically impair physician decision-making. Providing physicians with stress-modulating techniques is essential to optimizing clinical performance*.

Study investigators recruited eligible participants in January 2020 during each EM program’s weekly resident educational conference. During the recruitment session, the investigator conducted an overview of the study with the eligible participants and obtained consent for participation in the study. At that time subjects were randomized into intervention or control groups in an alternating fashion based on last name. Due to lower than anticipated attendance at residency conferences on the days of enrollment, residents were offered the opportunity to enroll into the control group on the day of the simulation assessment. In addition, some initially enrolled subjects were not present on the day of the simulation assessment due to conflicts with rotations and therefore were not included in the data collection.

### Study Protocol

At the time of study enrollment, each resident completed a pre-intervention survey that assessed perceptions about the incorporation of mental skills training and stress inoculation principles into residency training as well as prior exposure to these techniques (Supplemental Material).

During the recruitment session, faculty study investigators provided a 20-minute interactive, didactic session to the intervention group about the effects of acute stress on performance, specific mental skills to mitigate the effects of stress, and the application of these skills to high-stress clinical scenarios. The mental skills training was based on the “Breathe, Talk, See, Focus” (BTSF) approach to performance under pressure. The BTSF tool is a memory aid and training primer to help individuals rapidly employ mental skills proven effective in non-medical, high-stress domains.^21^,^22^ The tool itself consists of a four-step technique: 1) Breathe: Introduction to the concept of a ritualized form of breathing, such as “box breathing”; 2) Talk: Positive self-talk, recited with intention and repeated frequently; 3) See: Visualization of successful completion of a task; and 4) Focus: Use of a cue word to turn on selective attention.^21^ During the lecture itself, residents were introduced to the BTSF tool and also had the opportunity to practice using the tool as a group. Participants in the intervention group were strongly encouraged to review the BTSF tool and to attempt to implement these mental skills in their own clinical practice.

Each spring, all PGY-2 residents in Chicago area EM programs participate in an annual city-wide simulation assessment. While the assessment is based on the ACGME milestones and residents are given feedback on their performance, no participating residency uses the information to impact a resident’s standing, and this information is disclosed to the residents prior to participation. This study was designed to coincide with this event. Described in detail elsewhere, the simulation assessment consists of two procedure assessments and two high-fidelity, mannequin-based critically ill patient scenarios.^23^ During each of the four assessments, the PGY 2-resident is observed by a faculty member from a different residency program using a dichotomous checklist containing essential management actions corresponding to EM milestones. The PGY-2 residents are not informed of the content of the cases prior to participation in the assessment. One of the critically ill patient-simulation cases served as the study assessment. Participants were not aware of which case served as the study assessment.

Upon arrival for the PGY-2 simulation assessment in February 2020, residents in both the intervention and control groups completed a STAI-6 survey. The STAI-6 is a psychological inventory assessing anxiety about an event (state anxiety) and anxiety level as a personal characteristic (trait anxiety). While anxiety and stress are not synonymous, the inventory has been used in prior research as a surrogate for acute stress response.^24^ Both intervention and control group residents wore Polar H10 heart rate monitors (Polar Electro Oy, Kempele, Finland). Prior research has demonstrated correlations between the STAI-6 and HR and cortisol levels as well as a correlation between the STAI-6 and HRV during intubation attempts.^25^,^26^ Mefford et al also used HRV as an outcome measure in the evaluation of stress-modifying interventions and demonstrated that HRV may serve as an index of autonomic arousal.^26^ Similarly, Kim et al concluded that neurobiologic evidence suggests that HRV can be used as an objective measure of psychological stress.^27^ Pre-simulation physiologic parameters of HR and HRV were measured for a duration of five minutes using the Elite HRV application (Elite HRV, Asheville, NC) installed on the iPod Touch, 7^th^ generation (Apple Inc., Cupertino, CA).

Immediately prior to the start of the assessment case (and after pre-simulation HR and HRV were measured), residents randomized to the intervention group were provided a five-minute refresher on stress inoculation techniques and particularly in BTSF. The HRV and HR recordings were initiated five minutes prior to the assessment case and were recorded continuously until the case concluded. Biometric data was measured before, during, and after the simulation case. The following variables were recorded for each subject: HR (mean); minimum HR (lowest measured HR value); and maximum HR (highest measured HR value); HRV (mean). We calculated relative changes in all variables between baseline and during the simulation case.

As soon as the assessment case concluded, residents completed a second STAI-6 assessment. Upon completion of the PGY-2 simulation assessment, all residents completed the post-intervention survey (Supplemental Material).

## RESULTS

### Characteristics of Study Subjects

Among seven EM residency programs, there were 92 eligible PGY-2 EM residents. Ultimately, 47 residents underwent randomization after informed consent at the time of initial study enrollment. On the day of the PGY-2 simulation assessment, an additional 23 residents were consented for participation in the control group. Nine residents who initially were enrolled did not ultimately complete the study (six control and three intervention): three did not participate in the simulation assessment at all, one did not complete the surveys, and five declined to participate in the study. In total, 61 residents participated in the study, including 25 residents in the intervention group and 36 in the control group. There were no significant differences in age, gender, or ethnicity between the control and intervention groups (Table 1).

### Primary Results

The change in the pre- and post-resuscitation STAI-6 scores was not different between groups (−1.7 intervention, 0.4 control; *p* = 0.38), and there were no significant differences in mean pre- and post-resuscitation STAI-6 scores between groups (Table 2). In the control group, the mean STAI-6 scores pre- and post-resuscitation were 40 (standard deviation [SD] 6.6) and 41 (SD 5.9). In the intervention group, the mean STAI-6 scores pre- and post-resuscitation were 41.33 (SD 10.54) and 39.6 (SD 9.73). For reference, scores on the STAI-6 range from 20–80 with higher scores indicating more anxiety.

### Secondary Results

There were no significant differences in biometric data between groups (Table 3). The mean maximum HR during simulation was 133 in the control group and 136 in the intervention group (*p* = 0.97). The mean minimum HR during simulation was 70 in the control group and 71 in the intervention group (*p* = 0.76). There was no significant difference in mean HRV between groups (−3.8 millisecond [ms] intervention, −3.8 ms control; *p* = 0.58).

We found no significant group differences in the pre-intervention survey (Table 4). There were, however, significant differences in the post-intervention survey (Table 4). In response to the question “How relevant is the topic of stress inoculation to the resident physician?” 91% of the intervention group responded “very relevant” compared to 26% of the control group (*p* <0.01). In response to the question “How important is it to include education about stress inoculation topics in residency training?” 75% of the intervention group responded “very important” compared to 28% of the control group (*p* <0.01).

## DISCUSSION

In this multicenter, prospective educational trial of mental skills training for PGY-2 EM residents we found no demonstrable differences in subjective or objective measures of stress responses between the intervention and control groups. Nevertheless, there were statistically significant differences between the groups on the post-intervention surveys regarding resident perceptions of the importance of mental skills and stress-inoculation training in EM residencies. These differences were present only on the post-intervention survey, indicating that EM residents appreciated the value of this program only after exposure to this training.

Multiple prior studies have demonstrated the deleterious effects of acute stress on physician decision-making, physical performance, and performance during simulations.^2^–^5^ Relatively few studies have directly investigated the role of mental skills training and stress inoculation in mitigating these effects. A 2016 study of surgery trainees examined the impact of a mental skills curriculum on subjective stress.^24^ A study of novice PGY-1 EM residents demonstrated an association between perceived stress and anxiety and biometric data (HRV) when performing endotracheal intubations on live patients.^26^ To the best of our knowledge, this is the first study to investigate the effects of mental skills training on EM resident-perceived stress and anxiety as well as biometric data during a high-stress, simulated resuscitation. While our study failed to find significant differences between the groups in the self-reported and biometric data, the measurement of these parameters in the setting of a simulated resuscitation during which a group of subjects employed a mental skills technique was novel.

There are several possible explanations for the lack of significant differences in the STAI-6 and biometric data between the intervention and control groups. The intervention group received the initial training lecture approximately one month prior to the simulation assessment, and with the exception of the five-minute refresher prior to the simulation assessment there were no formal intervening reinforcements of the technique with the subjects. While the intervention subjects were encouraged to practice the BTSF tool during the month between the initial introduction and the simulation, utilization was not tracked. The lack of deliberate practice and delay from the initial exposure could have attenuated the benefits obtained from the introductory lecture, and decreased STAI-6, biometric, and performance data differences between the groups. It is also possible that the BTSF model is an ineffective method of performance enhancement under stress in EM residents. This seems less likely, however, as mental skills training has been shown to enhance performance and coping in stressful situations in other elite performance realms such as aviation, law enforcement, military special forces, professional athletics, and surgery, and that the BTSF model was developed by drawing on elements of similar effective paradigms.^12^–^16^,^21^

Our tested residents were in their second year of residency and may have already learned methods with which to manage stress in acute resuscitation scenarios based on either former didactic exposure or clinical experience. It is also possible that more of a stress effect would be demonstrated in a real-life scenario rather than a simulated one. Finally, to detect a 20% difference between groups we anticipated needing to enroll 36 subjects into each group. Due to conflicts impeding ability to participate, the intervention group only had 25 residents, which may have decreased our ability to detect a difference between the groups.

Future studies should focus on longitudinal investigations of mental skills training and stress-inoculation programs in which the mental performance tools are periodically reinforced prior to study assessment. Studies could also examine long-term resident perceptions of mental skills and stress-inoculation curricula, as well as resident perceptions of their own performance after being exposed to these topics. Finally, wearable technology such as the HR monitors used in this study could be used to measure biometric data during in vivo resuscitations or other high-stress medical scenarios.

## LIMITATIONS

There are several important limitations to this study. Foremost, initiatives to change behavior often require substantial time investment, training, and practice. While our stress response intervention was designed to be brief and easily implemented, the lack of directed and longitudinal exposure to the concepts likely affected the results. We also used the STAI-6 as a measure of acute stress response. While this has been previously used in other studies assessing similar stress response, it has not been independently validated in this utilization. In addition, although this was a multicenter trial, the study population was a convenience sample of PGY-2 residents in one specific geographic area. Furthermore, while subjects receiving the mental skills training were encouraged to use the techniques learned in the didactic session during the weeks preceding the simulation assessment, overall utilization was likely variable and not measured.

There were also a number of subject-specific, non-controlled confounders that may have impacted the biometric data, including stimulant ingestion (eg coffee, energy drinks), prescribed medications, sleep quality/duration in the preceding evening, and nutritional intake. Additionally, while the pre-simulation biometric data was gathered prior to the subjects participating in any simulated cases, it is possible that the subjects were anticipating the simulations and therefore were in a heightened physiologic state. Furthermore, the pre-simulation biometric data was collected for five minutes, which may not have been enough time to establish a completely accurate physiologic baseline for each participant. These factors may have confounded the biometric data.

We also did not examine resident performance in the simulated scenario as an outcome in the study. There could be an unexamined difference in stress response based on performance that was not determined. Finally, there were a few discrepancies in the biometric data (ie, a record heart rate in the mid-200s) that did not make physiologic sense and may have been due to artifact from the HR monitors and the biometric data-aggregation application. Such discrepancies may have skewed the biometric data.

## CONCLUSION

A brief, didactic mental skills training intervention did not demonstrate significant differences in subjective or objective measures of stress responses in EM residents during a simulated resuscitation. Residents in the intervention group were more likely to rate mental skills training as relevant and important. Future investigations involving comprehensive, longitudinal stress inoculation curricula are warranted.

## Supplementary Information



## Figures and Tables

**Table 1:** Subject demographic data.

	Intervention	Control	p -value
Experience			
Military	2 (15.4%)	1 (8.3%)	1.0
Law enforcement	0 (0.0%)	0 (0.0%)	N/A
Firefighting	1 (7.7%)	1 (8.3%)	1.0
Aviation	2 (15.4%)	2 (16.7%)	1.0
Sports	7 (53.9%)	6 (50.0%)	0.85
Arts	5 (38.5%)	4 (33.3%)	0.79
Age			
20–25	0 (0.0%)	0 (0.0%)	
25–30	15 (62.5%)	27 (77.1%)	
30–35	8 (33.3%)	6 (17.1%)	
>35	1 (4.2%)	2 (5.7%)	0.33
Gender			
Male	18 (75.0%)	22 (62.9%)	
Female	6 (25.0%)	13 (37.1%)	0.33
Race			
Black	0 (0.0%)	4 (11.1%)	
White	15 (62.5%)	16 (44.4%)	
Latinx	2 (8.3%)	1 (2.8%)	
Asian	6 (25.0%)	4 (11.1%)	
African	0 (0.0%)	0 (0.0%)	
Middle Eastern	1 (4.2%)	0 (0.0%)	
Native American/Inuit	0 (0.0%)	0 (0.0%)	
Pacific Islander	0 (0.0%)	0 (0.0%)	
Other	0 (0.0%)	11 (30.6%)	<0.01

* *P*-value <0.05 indicates significance.

** % is based on the number of individuals who answered each question.

**Table 2:** Comparisons of mean State-Trait Anxiety Inventory-6 scores between control and intervention groups.

	Intervention n = 25	Control n = 36	*p* -value
Pre-case STAI-6 score, mean ± SD	41±11	40.2 ±6.6	0.13
Post-case STAI-6 score, mean ± SD	40 ±9.7	41 ±5.9	0.83
Change in STAI-6 score, mean ± SD	−1.7 ±3.3	0.4 ±6.6	0.38

*STAI-6*, six-item short form of the Spielberger State-Trait Anxiety Inventory; *SD*, standard deviation.

**Table 3:** Comparison of mean biometric data between control and intervention groups.

	Intervention n = 25	Control n = 36	*p* -value
Baseline HRV, ms (mean ± SD)	55 ±8.5	55 ±9.0	0.94
Change in HRV, ms (mean ± SD)	−3.8 ±8.7	−3.8 ±10	0.58
Baseline HR, bpm (mean ± SD)	83 ±10	87 ±11	0.11
Baseline maximum HR (mean ± SD)	118 ±36	131 ±44	0.06
Max HR during simulation (mean ± SD)	136 ±42	133 ±35	0.97
Change in maximum HR (mean ± SD)	18 ±29	1.8 ±48	0.29
Baseline minimum HR (mean ± SD)	61 ±13	64 ±11	0.38
Minimum HR during simulation (mean ± SD)	71 ±61	70 ±17	0.76
Change in minimum HR (mean ± SD)	8.7 ±11	5.4 ±14	0.46

*HRV*, heart rate variability; *ms*, millisecond; *SD*, standard deviation; *HR*, heart rate; *bpm*, beats per minute.

**Table 4:** Comparisons of pre- and post-intervention survey responses*.

	Control pre-study	Intervention pre-study	Control post-study	Intervention post-study
How valuable would it be to incorporate a formal stress inoculation curriculum into EM residency training?				
Very valuable, n (%)	6 (40%)	3 (18%)	10 (28%)	18 (75%)
Somewhat valuable	6 (40%)	6 (35%)	14 (39%)	4 (17%)
Neutral	3 (20%)	6 (35%)	11 (31%)	1 (4.2%)
Not valuable	0 (0%)	2 (12%)	1 (2.8%)	1 (4.2%)
How relevant is the topic of stress inoculation to the EM resident physician?				
Very valuable, n (%)	7 (47%)	6(35%)	9 (26%)	19 (91%)
Somewhat valuable	6 (40%)	6 (35%)	14 (40%)	2 (13%)
Neutral	2 (13%)	4 (24%)	12 (34%)	0 (0%)
Not valuable	0 (0%)	1 (5.9%)	0 (0%)	0 (0%)

* % is based off the number of individuals who answered each question.

*EM*, emergency medicine.

## References

[1] Ericsson KA (2015). Acquisition and maintenance of medical expertise: a perspective from the expert-performance approach with deliberate practice. Acad Med.

[2] Cumming SR, Harris LM (2001). The impact of anxiety on the accuracy of diagnostic decision-making. Stress and Health.

[3] Harvey A, Bandiera G, Nathens AB, LeBlanc VR (2021). Impact of stress on resident performance in simulated trauma scenarios. J Trauma Acute Care Surg.

[4] Leblanc VR, Regehr C, Tavares W (2012). The impact of stress on paramedic performance during simulated critical events. Prehosp Disaster Med.

[5] Hunziker S, Semmer NK, Tschan F (2012). Dynamics and association of different acute stress markers with performance during a simulated resuscitation. Resuscitation.

[6] Driskell JE, Salas E (1996). Stress and Human Performance.

[7] Kahneman D (2011). Thinking, Fast and Slow.

[8] Leblanc VR (2009). The effects of acute stress on performance: implications for health professions education. Acad Med.

[9] Keinan G, Friedland N, Driskell JE, Salas E (1996). Training effective performance under stress: queries, dilemmas, and possible solutions. Stress and Human Performance.

[10] Saunders T, Driskell JE, Johnston JH (1996). The effects of stress inoculation training on anxiety and performance. J Occup Health Psychol.

[11] Johnston JH, Cannon-Bowers JA, Driskell JE, Salas E (1996). Training for stress exposure. Stress and Human Performance.

[12] McCrory P, Cobley S, Marchant P (2013). The effect of psychological skills training on self-regulation behavior, self-efficacy, and psychological skill use in military pilot trainees. Military Psych.

[13] Selder D, Burnett K, Nideffer R (1989). Psychological factors associated with the successful completion of basic underwater demolition SEAL training. US Navy Technical Report.

[14] Le Scanff C, Taugis J (2002). Stress management for police special forces. J of App Sport Psych.

[15] Guenthner SV, Hammermeister J, Burton D, Keller L (2010). Smoke and mirrors or wave of the future? Evaluating a mental skills training program for elite cross-country skiers. J of Sport and Behav.

[16] Rao A, Tait I, Alijani A (2015). Systematic review and meta-analysis of the orle of mental training in the acquisition of technical skills in surgery. Am J Surg.

[17] Bond WF, Spillane L (2002). The use of simulation for emergency medicine resident assessment. Acad Emerg Med.

[18] Piquette D, Tarshis J, Sinuff T (2014). Impact of acute stress on resident performance during simulated resuscitation episodes: a prospective randomized cross-over study. Teach Learn Med.

[19] Tluczek A, Henriques JB, Brown RL (2009). Support for the reliability and validity of a six-item state anxiety scale derived from the State-Trait Anxiety Inventory. J Nurs Meas.

[20] Marteau TM, Bekker H (1992). The development of a six-item short-form of the state scale of the Spielberger State-Trait Anxiety Inventory (STAI). Br J Clin Psychol.

[21] Lauria MJ, Gallo IA, Rush S (2017). Psychological skills to improve emergency care providers’ performance under stress. Ann Emerg Med.

[22] Weingart S (2018). EMCrit RACC Podcast 220– Beat the Stress Fool (BtSF) with Mike Lauria – Just in Time Performance-Enhancing Psychological Skills.

[23] Salzman DH, Watts H, Williamson K (2018). A multicenter collaboration for simulation-based assessment of ACGME milestones in emergency medicine. Simul Healthc.

[24] Anton NE, Howley LD, Pimentel M (2016). Effectiveness of a mental skills curriculum to reduce novices’ stress. J Surg Res.

[25] Arora S, Aggarwal R, Moran A (2011). Mental practice: effective stress management training for novice surgeons. J Am Coll Surg.

[26] Mefford JM, Kahle S, Gupta S (2019). Heart rate variability and acute stress among novice airway managers. AEM Educ Train.

[27] Kim H-G, Cheon E-J, Bai D-S (2018). Stress and heart rate variability: a meta-analysis and review of the literature. Psychiatry Investig.

