# Supplementary figures and images for: Effects of Brief Mental Skills Training on Emergency Medicine Residents’ Stress Response During a Simulated Resuscitation: A Prospective Randomized Trial
Source: West J Emerg Med. 2022 Jan 3;23(1):79–85. doi: 10.5811/westjem.2021.10.53892 (PMC8782128; doi:10.5811/westjem.2021.10.53892)

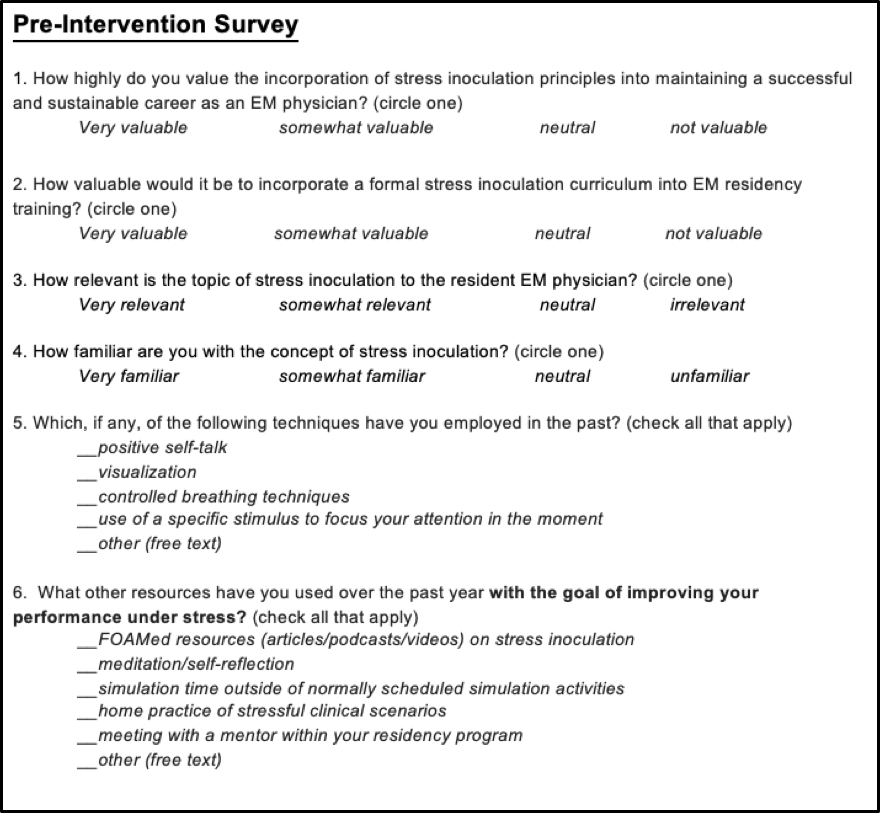

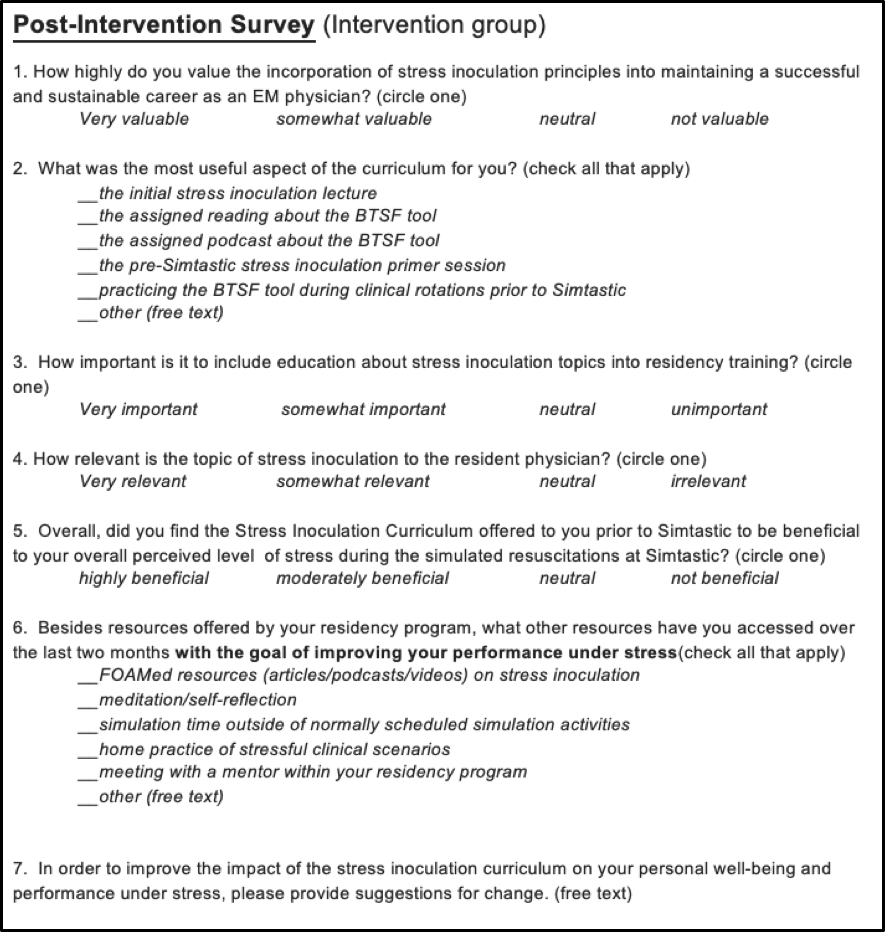

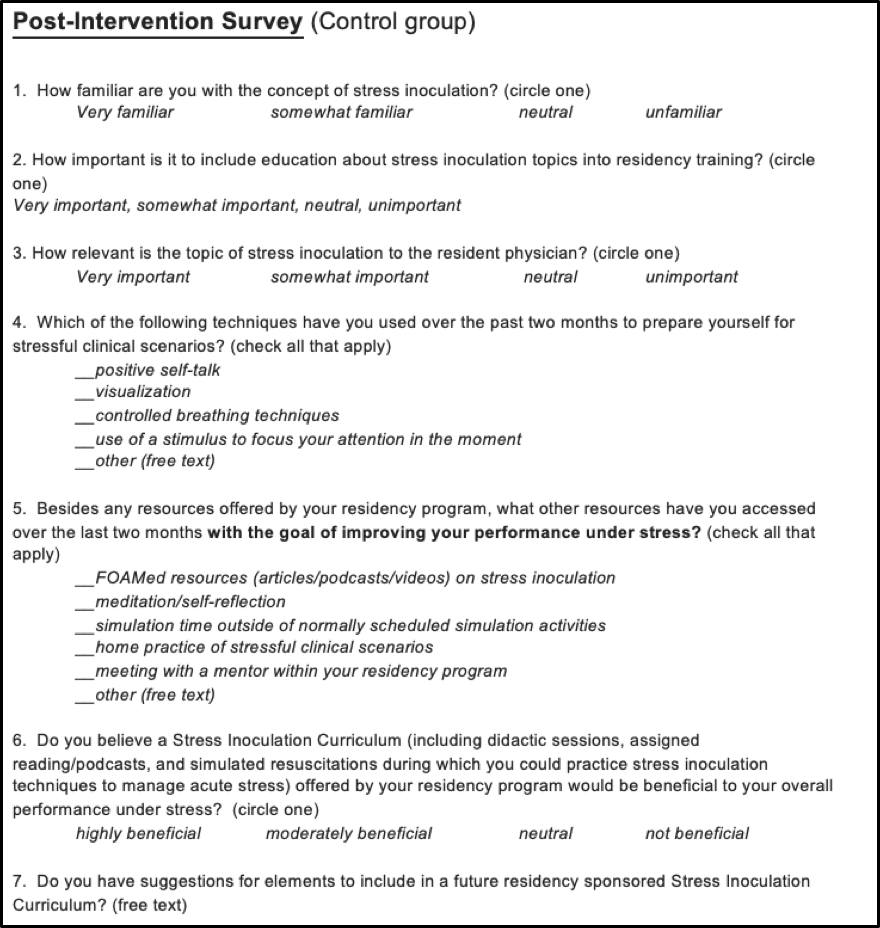

Supplement: Supplementary file 1 [file wjem-23-79-s001.docx]
